# Soil Microbiomes Associated with a Novel Perennial Grain Cultivated under Temperate Agricultural Conditions

**DOI:** 10.1128/mra.00204-23

**Published:** 2023-06-26

**Authors:** Tess Noble Strohm, Shannon Ryan, Carolina Oliveira de Santana, M. Eli Dueker, Gabriel G. Perron

**Affiliations:** a Department of Environmental and Urban Studies, Bard College, Annandale-on-Hudson, New York, USA; b Center for the Environmental Sciences and Humanities, Bard College, Annandale-on-Hudson, New York, USA; c Center for Genomics and Systems Biology, New York University, New York, New York, USA; d Department of Biological Sciences, State University of Feira de Santana, Feira de Santana, Bahia, Brazil; University of Maryland School of Medicine

## Abstract

A perennial wheatgrass called Kernza perennial grains was developed by the Land Institute to harness the benefits of perenniality on soil health in a commercial farming system. This study compared bacterial and fungal soil microbiomes surrounding 1-year-old Kernza, 4-year-old Kernza, and 6-week-old winter wheat in Hudson Valley, New York.

## ANNOUNCEMENT

Soil bacteria and fungi play essential roles in the soil ecosystem (1, 2). Bacteria assist in biogeochemical cycles and soil fertility, and fungi can improve growth, stress tolerance, and disease resistance in plants (3, 4). However, conventional farming practices, such as annual plants, can harm soil microbial communities (5). The perennial wheatgrass cultivar Kernza was developed by the Land Institute for the benefits they provide (6). Perennials provide a year-round carbon source to microbial communities, do not require tilling that may damage fungal networks, and can outcompete weeds without pesticide use (7). Here, we investigated the microbial diversity of soil surrounding a cultivar of Kernza intended for Hudson Valley, NY, at different ages, and an annual wheat crop grown under similar soil conditions.

**FIG 1:**
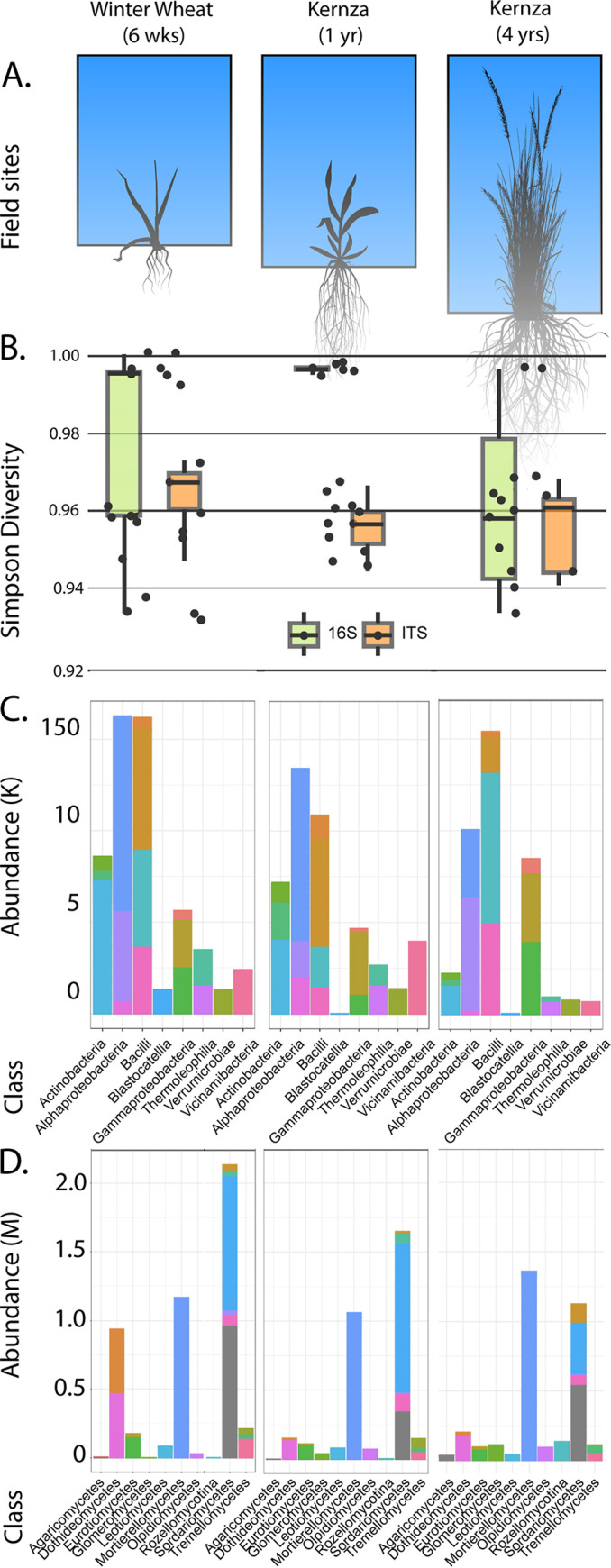
(A) Schematic representation of studied fields. From right to left: 6-week-old winter wheat (Field 20), 1-year-old Kernza (Field 3) and 4-year-old Kernza (Field 8). (B) Simpson diversity indices for prokaryotic and fungal communities at each site. (C) Prokaryotic classes with highest abundance in the data set. (D) Fungal classes with highest abundance in the data set.

On November 4, 2020, we collected 10 5-cm deep soil samples randomly selected from three crops, as follows: (i) field 3 (41°54′38″, N 74°5′30″ W), 1-year-old Kernza; (ii) field 8 (41°54′42″ N, 74°5′25″ W), 4-year-old Kernza; and (iii) field 20 (41°54′55″ N, 74°5′15″ W), 6-week-old annual winter wheat (Fig. 1A). The samples were collected ~5 cm from the base of each plant. DNA was extracted using the Zymo quick-DNA fecal/soil microbe miniprep kit. Sequencing of 16S rRNA and ITS amplicons was performed at Wright Labs (Huntingdon, PA) using the Earth Microbiome Project protocol (8). Briefly, the 515F and 806R primers were used for amplifying the V4 region of the 16S rRNA gene (8) while ITS1f and ITS2 primers were used to amplify the ITS gene (9). PCR products for the 16S rRNA and ITS amplicons were pooled separately and purified on a 2% agarose gel using the Qiagen gel extraction kit; approximately 200 to 600 bp for the ITS amplicons and ~385 bp for the 16S rRNA amplicons were excised. After a quality check, amplicon libraries were sequenced using the Illumina MiSeq v2 paired-end sequencing platform (2 × 250-bp reads) for 16S rRNA and single reads for ITS with 20% PhiX spike-in.

Raw reads were processed using DADA2 v1.18 (10) using default parameters (except for 16S rRNA, denoise-paired, -p-time-left-f 0 pp-time-left-r 0 -p-trunc-len-f 250 -p-trunc-len-r 250; ITS, denoised-single, -p-trunc-len 150) as implemented in R v3.6.2 (11). Due to read counts below 5,000 following preprocessing, two samples from field 3 and three samples from field 8 were removed from the 16S rRNA analysis. Three samples from field 3 and three samples from field 8 were removed from the ITS analysis. We found the highest Simpson diversity of prokaryotes in field 3, followed by field 20 and field 8 (Fig. 1B). Alternatively, field 20 had the highest fungal diversity, followed by field 8, and field 3 (Fig. 1B). Taxonomic assignment using the Silva database v138.1 (12) revealed a predominance of *Alphaproteobacteria* in fields 3 and 20 and *Bacilli* in field 8 (Fig. 1C). Using the UNITE database (13), we found a predominance of the fungal groups *Sordariomycetes* in field 3 and field 20 and *Mortierellomycetes* in field 8 (Fig. 1D). Visualization was performed using the phyloseq package v1.30.0 (14).

### Data availability.

All data are available through NCBI SRA Project PRJNA940179. Individual accessions for each sample can be found in Table 1.

**TABLE 1:** Summary of data

File name by gene	SRA no.	Avg read length (bp)	No. of reads
16S			
F3_1_S1_R1_001.fastq.gz	SRR23715909	264.06	9,882
F3_1_S1_R2_001.fastq.gz	SRR23715909	256.958	9,882
F3_2_S44_R1_001.fastq.gz	SRR23715908	264.467	116,003
F3_2_S44_R2_001.fastq.gz	SRR23715908	264.472	116,003
F3_3_S2_R1_001.fastq.gz	SRR23715897	256.386	25,200
F3_3_S2_R2_001.fastq.gz	SRR23715897	252.993	25,200
F3_4_S45_R1_001.fastq.gz	SRR23715875	266.675	192,652
F3_4_S45_R2_001.fastq.gz	SRR23715875	272.965	192,652
F3_5_S46_R1_001.fastq.gz	SRR23715886	265.77	146,316
F3_5_S46_R2_001.fastq.gz	SRR23715886	274.355	146,316
F3_6_S47_R1_001.fastq.gz	SRR23715865	267.604	196,535
F3_6_S47_R2_001.fastq.gz	SRR23715865	276.567	196,535
F3_7_S48_R1_001.fastq.gz	SRR23715855	266.636	94,302
F3_7_S48_R2_001.fastq.gz	SRR23715855	266.024	94,302
F3_8_S49_R1_001.fastq.gz	SRR23715854	266.807	126,980
F3_8_S49_R2_001.fastq.gz	SRR23715854	265.183	126,980
F3_9_S50_R1_001.fastq.gz	SRR23715901	266.914	203,644
F3_9_S50_R2_001.fastq.gz	SRR23715901	277.273	203,644
F3_10_S3_R1_001.fastq.gz	SRR23715852	243.645	16,220
F3_10_S3_R2_001.fastq.gz	SRR23715852	256.009	16,220
F8_1_S4_R1_001.fastq.gz	SRR23715907	264.549	21,686
F8_1_S4_R2_001.fastq.gz	SRR23715907	260.7	21,686
F8_2_S51_R1_001.fastq.gz	SRR23715906	263.895	133,591
F8_2_S51_R2_001.fastq.gz	SRR23715906	277.224	133,591
F8_3_S52_R1_001.fastq.gz	SRR23715905	267.541	79,074
F8_3_S52_R2_001.fastq.gz	SRR23715905	283.73	79,074
F8_4_S53_R1_001.fastq.gz	SRR23715904	249.036	36,329
F8_4_S53_R2_001.fastq.gz	SRR23715904	252.733	36,329
F8_5_S54_R1_001.fastq.gz	SRR23715903	254.277	36,120
F8_5_S54_R2_001.fastq.gz	SRR23715903	252.578	36,120
F8_6_S5_R1_001.fastq.gz	SRR23715902	257.879	10,455
F8_6_S5_R2_001.fastq.gz	SRR23715902	264.921	10,455
F8_7_S6_R1_001.fastq.gz	SRR23715901	267.34	6,184
F8_7_S6_R2_001.fastq.gz	SRR23715901	267.758	6,184
F8_8_S55_R1_001.fastq.gz	SRR23715900	252.63	54,254
F8_8_S55_R2_001.fastq.gz	SRR23715900	256.987	54,254
F8_9_S7_R1_001.fastq.gz	SRR23715899	264.04	39,312
F8_9_S7_R2_001.fastq.gz	SRR23715899	266.646	39,312
F8_10_S8_R1_001.fastq.gz	SRR23715898	261.352	10,605
F8_10_S8_R2_001.fastq.gz	SRR23715898	274.894	10,605
WWF20_1_S82_R1_001.fastq.gz	SRR23715896	269.212	105,417
WWF20_1_S82_R2_001.fastq.gz	SRR23715896	282.634	105,417
WWF20_2_S56_R1_001.fastq.gz	SRR23715895	247.004	31,813
WWF20_2_S56_R2_001.fastq.gz	SRR23715895	254.867	31,813
WWF20_3_S83_R1_001.fastq.gz	SRR23715868	268.1	138,032
WWF20_3_S83_R2_001.fastq.gz	SRR23715868	273.935	138,032
WWF20_4_S57_R1_001.fastq.gz	SRR23715869	255.623	84,997
WWF20_4_S57_R2_001.fastq.gz	SRR23715869	253.905	84,997
WWF20_5_S58_R1_001.fastq.gz	SRR23715870	261.709	93,141
WWF20_5_S58_R2_001.fastq.gz	SRR23715870	261.585	93,141
WWF20_6_S84_R1_001.fastq.gz	SRR23715892	261.169	135,574
WWF20_6_S84_R2_001.fastq.gz	SRR23715892	275.944	135,574
WWF20_7_S59_R1_001.fastq.gz	SRR23715893	262.716	125,621
WWF20_7_S59_R2_001.fastq.gz	SRR23715893	262.394	125,621
WWF20_8_S85_R1_001.fastq.gz	SRR23715894	261.642	108,268
WWF20_8_S85_R2_001.fastq.gz	SRR23715894	275.377	108,268
WWF20_9_S60_R1_001.fastq.gz	SRR23715873	272.181	100,222
WWF20_9_S60_R2_001.fastq.gz	SRR23715873	270.388	100,222
WWF20_10_S86_R1_001.fastq.gz	SRR23715874	269.891	133,328
WWF20_10_S86_R2_001.fastq.gz	SRR23715874	278.928	133,328
ITS			
F3_3_S15_R1_001.fastq.gz	SRR23715877	239.945	689,211
F3_4_S16_R1_001.fastq.gz	SRR23715878	242.617	308,924
F3_5_S17_R1_001.fastq.gz	SRR23715879	244.314	282,022
F3_6_S18_R1_001.fastq.gz	SRR23715880	245.544	696,105
F3_7_S19_R1_001.fastq.gz	SRR23715881	244.641	1,156,088
F3_9_S21_R1_001.fastq.gz	SRR23715872	240.634	737,658
F3_10_S22_R1_001.fastq.gz	SRR23715884	241.7	917,528
F8_4_S26_R1_001.fastq.gz	SRR23715889	243.53	803,607
F8_5_S27_R1_001.fastq.gz	SRR23715890	246.034	404,244
F8_6_S28_R1_001.fastq.gz	SRR23715891	243.207	901,998
F8_7_S29_R1_001.fastq.gz	SRR23715872	242.061	452,413
F8_8_S30_R1_001.fastq.gz	SRR23715871	239.402	700,246
F8_9_S31_R1_001.fastq.gz	SRR23715851	241.192	1,775,913
F8_10_S32_R1_001.fastq.gz	SRR23715867	241.23	485,378
WWF20_1_S33_R1_001.fastq.gz	SRR23715866	236.082	1,803,118
WWF20_2_S34_R1_001.fastq.gz	SRR23715866	236.509	1,411,079
WWF20_3_S35_R1_001.fastq.gz	SRR23715863	242.746	152,795
WWF20_4_S36_R1_001.fastq.gz	SRR23715862	238.164	223,491
WWF20_5_S37_R1_001.fastq.gz	SRR23715861	236.827	1,813,664
WWF20_6_S38_R1_001.fastq.gz	SRR23715860	238.149	1,444,741
WWF20_7_S39_R1_001.fastq.gz	SRR23715859	237.337	1,664,740
WWF20_8_S79_R1_001.fastq.gz	SRR23715858	236.376	2,160,300
WWF20_9_S40_R1_001.fastq.gz	SRR23715857	236.811	1,995,189
WWF20_10_S41_R1_001.fastq.gz	SRR23715856	236.678	1,711,105

## References

[1] Frąc M, Hannula SE, Bełka M, Jędryczka M. 2018. Fungal biodiversity and their role in soil health. Front Microbiol 9:707. doi:10.3389/fmicb.2018.00707. 29755421 PMC5932366

[2] Mattoo R, Gowda M. 2022. Harnessing soil bacteria and their benefits for sustainable agriculture with changing climate. CABI Rev. doi:10.1079/cabireviews202217002.

[3] Hayat R, Ali S, Amara U, Khalid R, Ahmed I. 2010. Soil beneficial bacteria and their role in plant growth promotion: a review. Ann Microbiol 60:579–598. doi:10.1007/s13213-010-0117-1.

[4] Chen M, Arato M, Borghi L, Nouri E, Reinhardt D. 2018. Beneficial services of arbuscular mycorrhizal fungi—from ecology to application. Front Plant Sci 9:1270. doi:10.3389/fpls.2018.01270. 30233616 PMC6132195

[5] Crews TE, Carton W, Olsson L. 2018. Is the future of agriculture perennial? Imperatives and opportunities to reinvent agriculture by shifting from annual monocultures to perennial polycultures. Glob Sustain 1:e11. doi:10.1017/sus.2018.11.

[6] Peixoto L, Olesen JE, Elsgaard L, Enggrob KL, Banfield CC, Dippold MA, Nicolaisen MH, Bak F, Zang H, Dresbøll DB, Thorup-Kristensen K, Rasmussen J. 2022. Deep-rooted perennial crops differ in capacity to stabilize C inputs in deep soil layers. Sci Rep 12:5952. doi:10.1038/s41598-022-09737-1. 35396458 PMC8993804

[7] McKenna TP, Crews TE, Kemp L, Sikes BA. 2020. Community structure of soil fungi in a novel perennial crop monoculture, annual agriculture, and native prairie reconstruction. PLoS One 15:e0228202. doi:10.1371/journal.pone.0228202. 31999724 PMC6991957

[8] Walters W, Hyde ER, Berg-Lyons D, Ackermann G, Humphrey G, Parada A, Gilbert JA, Jansson JK, Caporaso JG, Fuhrman JA, Apprill A, Knight R. 2016. Improved bacterial 16S rRNA gene (V4 and V4-5) and fungal internal transcribed spacer marker gene primers for microbial community surveys. mSystems 1:e00009-15. doi:10.1128/mSystems.00009-15. PMC5069754 27822518

[9] Bokulich NA, Mills DA. 2013. Improved selection of internal transcribed spacer-specific primers enables quantitative, ultra-high-throughput profiling of fungal communities. Appl Environ Microbiol 79:2519–2526. doi:10.1128/AEM.03870-12. 23377949 PMC3623200

[10] Callahan BJ, McMurdie PJ, Rosen MJ, Han AW, Johnson AJA, Holmes SP. 2016. DADA2: high-resolution sample inference from Illumina amplicon data. Nat Methods 13:581–583. doi:10.1038/nmeth.3869. 27214047 PMC4927377

[11] R Core Team. (2021). R: a language and environment for statistical computing. R Foundation for Statistical Computing, Vienna, Austria. https://www.R-project.org/.

[12] Quast C, Pruesse E, Yilmaz P, Gerken J, Schweer T, Yarza P, Peplies J, Glöckner FO. 2012. The SILVA ribosomal RNA gene database project: improved data processing and Web-based tools. Acids Res 41:D590–D596. doi:10.1093/nar/gks1219. PMC3531112 23193283

[13] Kõljalg U, Larsson K-H, Abarenkov K, Nilsson RH, Alexander IJ, Eberhardt U, Erland S, Høiland K, Kjøller R, Larsson E, Pennanen T, Sen R, Taylor AFS, Tedersoo L, Vrålstad T, Ursing BM. 2005. UNITE: a database providing Web-based methods for the molecular identification of ectomycorrhizal fungi. New Phytol 166:1063–1068. doi:10.1111/j.1469-8137.2005.01376.x. 15869663

[14] McMurdie PJ, Holmes S. 2013. phyloseq: an R package for reproducible interactive analysis and graphics of microbiome census data. PLoS One 8:e61217. doi:10.1371/journal.pone.0061217. 23630581 PMC3632530

